# Malnutrition and Associated Risk Factors Among Disabled Children. Special Considerations in the Pediatric Surgical “Fragile” Patients

**DOI:** 10.3389/fped.2019.00086

**Published:** 2019-03-22

**Authors:** Gloria Pelizzo, Valeria Calcaterra, Carlo Acierno, Hellas Cena

**Affiliations:** ^1^Pediatric Surgery Department, Children's Hospital G. Di Cristina, ARNAS Civico-Di Cristina-Benfratelli, Palermo, Italy; ^2^Pediatric Unit, Department of Maternal and Children's Health, Fondazione IRCCS Policlinico San Matteo and Department of Internal Medicine, University of Pavia, Pavia, Italy; ^3^Laboratory of Dietetics and Clinical Nutrition, Department of Public Health, Neurosciences, Experimental and Forensic Medicine, University of Pavia, Pavia, Italy; ^4^Clinical Nutrition and Dietetics Service, Unit of Internal Medicine and Endocrinology, ICS Maugeri IRCCS, Pavia, Italy

**Keywords:** pediatric surgery, children, disability, health care, malnutrition

Surgery is one of the most stressful events that can trigger several inflammatory and catabolic pathways (1). An adequate nutritional status allows the body to react properly to this stressor and recover in a faster and more efficient way (1). On the other hand, malnutrition has been identified as a significant contributor correlated to a worse surgery outcome and to a higher prevalence of comorbidities and mortality (1, 2).

Nutrition and disability are intimately linked (3). Malnutrition can occur frequently in pediatric patients with neuro-physical impairment due to inappropriate dietary energy intake, oral motor dysfunction, increased nutrient losses, increased basal metabolic rate, and physical exertion (4, 5).

Poor nutrition, including insufficient dietary intake as a consequence of feeding difficulties and less frequently overnutrition due to energy imbalance, affects growth in children and impacts negatively on body composition, muscle strength, bone metabolism, as well as metabolic/endocrine profile, cardiovascular and respiratory systems, immune function, wound healing, all of which impair surgical outcome (3, 4, 6–9). A strong correlation between the severity of nutritional impairment and an increased risk of subsequent morbid events has been identified (10). This risk is increased during surgery, when stress response to surgery may also accentuate catabolic processes (3, 4, 7–9).

Disabled children may require surgery primarily for gastroesophageal reflux, gastrointestinal dismotilities, skeletal deformities or even neurolorogical, and cardiological comorbitities. In literature, poor nutritional status has been reported in nearly 40% of neurologically impaired (NI) children undergoing surgery (5). The prevalence of malnutrition is related to age and severity of neurological disability and it is not primarily determined by energy intake (5, 11); it also occurs in patients with adequate energy intake or even higher intakes than expected (5). Indeed it is well known that several factors, such as inflammation and hormonal imbalance may exert a negative impact on nutritional status. Macronutrients and micronutrients deficiencies have been described, including vitamin D, B12, and folate deficiencies, as well as biomarkers of impaired nutritional status such as prealbumin and plasma iron, reported in 50, 46.6/58, 27.9, and 19.5% of the children, respectively (5).

In this “fragile” pediatric population malnutrition is correlated to an unfavorable metabolic risk profile, including dyslipidemia, elevated transaminase levels, and mostly insulin resistance (IR). Indeed pathological serum levels of triglycerides, HDL-cholesterol and transaminase are reported in 27.2, 14.2, and 27.2%, respectively (5), as well as IR was observed in 50% of the patients, without any association to BMI, body composition or other MS components. The effects of childhood malnutrition on metabolic disorders are not yet fully elucidated. Unfulfilled energy and nutrients requirements as well as non-nutritional factors including restricted physical activity, long-term anticonvulsant drugs, chronic stress, inflammatory status, physiologic adaptive mechanism, organ impairment can lead to dysmetabolism (5, 12).

In individuals exposed to repeated or chronic stress, such as disability, the glucocorticoid (GC)-dependent stress-response negative feedback mechanism is impaired, leading to GC receptor resistance, and high systemic levels of molecular stress mediators, affecting the immune system and damaging multiple organs and tissues over time (13). A high cumulative physiological wear and tear, referred to allostatic load (AL) and considered a marker of chronic stress, has already been described in disabled children (14). AL is a preclinical marker of pathophysiological processes that can be considered a key mediator of adversity and negative health outcomes able to predict cardiovascular disease progression and mortality (14). More than 40% of the NI subjects showed high AL, significantly correlated with malnutrition (13) and potentially influencing outcome. Moreover, metabolic abnormalities and chronic stress are crucial factors for stress-induced hyperglycemia, which occur during stressful situations, such as surgery, increasing risk for infections and wound healing (5, 15–17) which in turn is related to acquired immune deficiency, due to poor nutritional status, common in children with disability (18).

Prolonged stress and metabolic disorders have also been associated with a less healthy cardiovascular system profile. Some conditions associated with intellectual or developmental disabilities in children remain highly correlated with cardiovascular risk factors in adulthood (19, 20). In disabled children, predictive cardiovascular signs are reported: hypertension was present in more than 15% of NI children and a epicardial fat measurement, considered an independent predictor of coronary atherosclerotic burden, was thicker than age-matched-controls (21).

In malnourished surgical patients awaiting major surgery, available data suggested that also body composition deviates from normality (18). All the patients who lose weight may be considered like having a “shrinking body cell mass surrounded by a growing sea of extracellular fluid” (18). These data have also been confirmed in NI surgical children, recording higher resistance, and lower reactance values, measured by Bioelectric Impedance Analysis (BIA), compared to normal weight young subjects (5) and extremely lower phase angle values, index of cell membrane integrity loss (22–24).

For this reason the malnourished surgical patients are usually intolerant of excessive salt and water loads having a tendency toward hyponatraemia, hypotonicity, and oedema (18). Additionally, the direct correlation of AL with fat mass is suggestive of greater metabolic and cardiovascular compromise due to the systematic inflammation that starts with an increase in silent inflammation in the adipose tissue (14). Moreover, an inverse correlation of AL with fat free mass, may be considered a predictive sign of decreased protection and increased fragility.

Respiratory function has also been shown to be affected in malnourished subjects, with a reduced capacity of adequate levels of ventilation sustainability due to the central nervous system and respiratory muscles inability (18), leading to a neural ventilatory drive impairment and inspiratory and expiratory muscular weakness (18), with difficult anesthesiological airways management.

Malnutrition, particularly protein energy malnutrition (PEM), contributes to the occurrence of osteoporotic fracture, by lowering bone mass and altering muscle strength. NI children are considered to be at high risk for bone fracture, also shared by altered muscle control, increased muscular tone, and diminished mineralization; lifetime fracture prevalence, between 6 and 12% has been reported in physically disabled children (25–27). These children have been known for osteopenia and low bone mineral density (28, 29) due in part to insufficient or no mobility at all, besides hypovitaminosis D, caused by hormonal factors, anticonvulsant therapy, low sun exposure, and poor nutrition (5, 25, 28). The fractures are either “spontaneous,” with no apparent history of injury, or occur with minimal trauma and are associated with a higher complication rate than fractures in healthy children (30). Preoperative mobilization and/or periods of immobilization due to surgery further exacerbate this problem (25, 27).

All these malnutrition-related consequences negatively impact surgical outcome, with an increased risk of post-operative pneumonia, surgical-site infection, sutures dehiscence requiring re-do surgery, sepsis, and should be considered as a measure for further studies with the aim to preserve health and/or reduce the risk of worsening the precarious conditions faced by these children. These data should be used to promote quality of life in at-risk disabled populations before, during, and after surgical procedures. Nutritional assessment must become a crucial part before performing surgical treatment in NI subjects (5).

This evaluation should start early immediately after the clinical diagnosis of disability, in all fragile children.

In order to facilitate health outcomes in severely disabled patients, before surgery an extensive panel of clinical and laboratory measurements should be performed to assess nutritional status, cumulative biological dysregulation and subclinical manifestations of disease. Anthropometric measurements of height, weight, calculation of BMI, pubertal stage, and arm muscle circumference, estimation of energy intake and expenditure; assessment of the lifestyle and mobility, as well as laboratory analysis of renal and hepatic function, lipids levels, serum proteins with shorter half-lives, transferrin and vitamins values, insulin resistance, and stress hormone levels; evaluation of preclinical cardiovascular signs and medications are recommended.

Disability clinically detectable in children has a significant higher morbidity and mortality risk and requires an early pediatric multidisciplinary approach including, endocrinologist, nutritionists, orthopedics, neurologists, and surgeons. Patients at higher risk of surgical stress and complications should be identified and submitted to preoperative nutritional support, which should be performed as soon as possible in pediatrics to prevent complications in case of surgery. The nutritional aspect should be carefully analyzed, focusing the attention on energy and nutrients requirement and the importance of an individualized nutritional approach should be acknowledged. Nutritional intervention need to be tailored on the singular case according to an accurate nutritional assessment including body composition analysis, laboratory tests, feeding history and difficulties, besides medical history as already proposed by Penagini et al. (6).

Care improvements in fragile children will help to protect their health, welfare, and survival as well as their quality of life.

It is up to the pediatric surgeon to reserve a special consideration of fragile patient before, during and in long term postoperative follow-up and to recommend a dedicated Enhanced Recovery After Surgery protocol (31).

## Author Contributions

GP, VC, and HC made a substantial contribution to the concept or design of the work and drafted the article or revised it critically for important intellectual content. CA made a contribution to the acquisition of data and drafted the article. All authors approved the version to be published.

### Conflict of Interest Statement

The authors declare that the research was conducted in the absence of any commercial or financial relationships that could be construed as a potential conflict of interest.
